# Severe strongyloidiasis: a systematic review of case reports

**DOI:** 10.1186/1471-2334-13-78

**Published:** 2013-02-08

**Authors:** Dora Buonfrate, Ana Requena-Mendez, Andrea Angheben, Jose Muñoz, Federico Gobbi, Jef Van Den Ende, Zeno Bisoffi

**Affiliations:** 1 Centre for Tropical Diseases (CTD), Sacro Cuore Hospital, Negrar, Verona, Italy; 2 Barcelona Centre for International Health Research (CRESIB) Hospital Clinic, Barcelona, Spain; 3 Department of Clinical sciences, Institute of Tropical Medicine, Antwerp, Belgium

**Keywords:** Strongyloidiasis, *Strongyloides*, Hyperinfection, Disseminated strongyloidiasis, Review

## Abstract

**Background:**

Strongyloidiasis is commonly a clinically unapparent, chronic infection, but immuno suppressed subjects can develop fatal disease. We carried out a review of literature on hyperinfection syndrome (HS) and disseminated strongyloidiasis (DS), in order to describe the most challenging aspects of severe strongyloidiasis.

**Methods:**

We conducted a structured search using PubMed to collect case reports and short case series on HS/DS published from 1991 to 2011. We restricted search to papers in English, Spanish, Italian and French. Case reports were classified as HS/DS according to given definitions.

**Results:**

Records screened were 821, and 311 were excluded through titles and abstract evaluation. Of 510 full-text articles assessed for eligibility, 213 were included in qualitative analysis. As some of them were short case series, eventually the number of cases analyzed was 244.

Steroids represented the main trigger predisposing to HS and DS (67% cases): they were mostly administered to treat underlying conditions (e.g. lymphomas, rheumatic diseases). However, sometimes steroids were empirically prescribed to treat signs and symptoms caused by unsuspected/unrecognized strongyloidiasis. Diagnosis was obtained by microscopy examination in 100% cases, while serology was done in a few cases (6.5%). Only in 3/29 cases of solid organ/bone marrow transplantation there is mention of pre-transplant serological screening. Therapeutic regimens were different in terms of drugs selection and combination, administration route and duration. Similar fatality rate was observed between patients with DS (68.5%) and HS (60%).

**Conclusions:**

Proper screening (which must include serology) is mandatory in high - risk patients, for instance candidates to immunosuppressive medications, currently or previously living in endemic countries. In some cases, presumptive treatment might be justified. Ivermectin is the gold standard for treatment, although the optimal dosage is not clearly defined in case of HS/DS.

## Background

Strongyloidiasis is a neglected condition caused by *Strongyloides stercoralis*, a soil – transmitted helminth mainly diffused in tropical and subtropical regions, but also present in small areas of low endemicity in temperate climates [1]. Most infected individuals are asymptomatic or may present intermittent symptoms, mostly affecting intestine (from mild abdominal pain, intermittent or persistent diarrhea to more severe conditions that can mimic inflammatory bowel disease), lungs (cough, wheezing and asthma, chronic bronchitis) and skin (pruritus, rash). Systemic symptoms such as weight loss and cachexia may also occur [2]. Immune suppressed subjects tend to develop hyperinfection syndrome (HS) and disseminated strongyloidiasis (DS), that are potentially fatal [3]. Therefore, it is mandatory to diagnose and treat the chronic infection, in order to prevent the life-threatening form. Unfortunately, the index of suspicion of health care providers seems to be low, especially in non-endemic countries [4]. Moreover, there are still gaps in knowledge regarding many aspects of the infection, such as diagnosis and treatment response [2].

Our aim was to systematically review case reports of severe strongyloidiasis, in order to outline the main features of hyperinfection and disseminated strongyloidiasis and the difficulties in their management.

## Methods

We carried out a systematic review of case reports/short case series published in PubMed from January 1991 to April 2011. We considered papers available in the following languages: English, Spanish, Italian, French.

The electronic search strategy was as follows: disease (strongyl*, anguillulose) AND severity of cases (disseminat*, hyperinfect*, severe, death, fatal, mortality) OR disease (strongyl*, anguillulose) AND associated conditions (tumor*, cancer, haematolog*, lymphom*, leukem*, leukaem*, neoplas*, malignan*, HTLV*, HIV, AIDS, hypogammaglobulinemia, rheumat*, “biological agents”, diabet*, transplant*, COPD, steroid*, glucocorticoid*, Immunosuppression [MeSH], Immunocompromised Host [MeSH]) and limiting the search to humans. Search was done on March 20th 2011.

Definitions used for case - inclusion [5]: - Dissemination: larvae found in any organ, other than the respiratory and the gastrointestinal tracts. Hyperinfection: infection confined to lungs and gastrointestinal tract, but signs/symptoms of severe diseases in relation to elevated number of larvae; in particular, necessity of intensive care, presence of sepsis/meningitis by enteric bacteria, death (without any other clear underlying cause).

## Results

### Data synthesis

**Figure 1:**
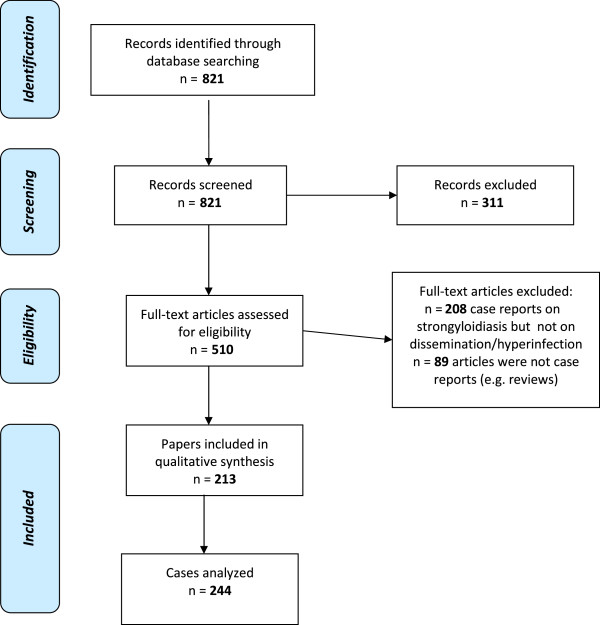
PRISMA flow chart: data collection and selection of studies.

Our search strategy permitted to identify 821 papers, of which 311 were excluded by title and abstract evaluation. Full-text papers were then assessed for eligibility according to the criteria outlined above. Among the 213 papers included, some were small case series, eventually the number of cases analyzed was 244 (Figure 1).

### Countries

Reports from highly endemic countries were 65/244 (27%), with India ( [6-23]), Argentina ( [24-28]), Brazil ( [29-39]) and Peru ( [40-42]) accounting for more than two thirds. Only four cases were reported from the whole of Africa [43-46], three of which in South Africa, a state where adequate diagnostic facilities are available. We collected 83/244 (34%) reports from North America (USA [47-109] and Canada [110-116]), 58/244 (24%) from Europe (Belgium [40,117], France [118-129], Germany [130,131], Greece [132-134], Italy [135-141], the Netherlands [142-145], Spain [146-155], Switzerland [156], UK [157-164]) and five (2%) from Oceania (Australia [165-168] and New Zealand [169]). In these areas of low/no endemicity, half of the patients were immigrants (70/146, 48%), while a few subjects were veterans (5/146, 3%) who presumably acquired the infection during military service in an endemic country. Other areas of low endemicity where cases have been reported are in Eastern Asia (21 cases, mostly from Japan ( [170-177]) and Taiwan ( [178-182])), the Arabian peninsula (nine cases, mostly from Kuwait ( [183,184]) and Qatar ( [185,186])) and Israel ( [187,188]) (three cases). Countries such as Iran ( [189]), Turkey ( [190]) and Venezuela ( [191]) that might be presumed at medium to high prevalence, account for only one case each.

### Triggers for development of HS/DS

According to the case definitions, 171 cases were classified as hyper infection and 73 cases as dissemination.

A high percentage of patients (67%: 164/244) were under corticosteroids: most of them presented clinical conditions causing immune suppression per-se or due to other related therapies (for instance leukemia, rheumatic conditions, transplant), as it is shown in Table 1. On the other hand, a few patients were taking steroids for eosinophilia and/or a specific symptoms caused by *S. stercoralis* itself (data reported in Table 1 too). A patient even underwent bone marrow transplant because of an unexplained eosinophilia misdiagnosed as “idiopathic hypereosinophilic syndrome” [81]; after receiving steroids and immunosuppressive therapy he developed HS (but only limited autopsy was performed, so we cannot rule out DS) and died.

**Table 1:** Patients under steroid treatment: reasons for prescription

**Condition**	**N (%)**	**References**
COPD/asthma/lung fibrosis	30 (18.3)	[48,49,52,57-59,68,99,101,118,121,123,128,137,146,153,180-183,185,187,188,192-196]
Leukemia/lymphoma	13 (7.9)	[9,17,23,25,37,47,56,98,111,126,162,186]
SLE	9 (5.5)	[41,64,66,86,151,176,197,198]
Rheumatoid arthtritis	4 (2.4)	[83,103,199,200]
IBD	6 (3.6)	[59,147,148,164,177,201]
Sarcoidosis	2 (1.2)	[65,132]
Cancer	8 (4.8)	[30,54,93,97,112,160,169,202]
Organ/bone marrow transplant	25 (15.2)	[21,25,29,31,39,48,51,54,60,70,71,74,76,81,87,88,90,92,94,142,145,150,184]
Glomerulonephritis/CRI	6 (3.6)	[16,18,20,129,130,154]
“Idiopatic” eosinophilia	3 (1.8)	[7]
Multiple myeloma/myelodisplasia	6 (3.6)	[72,185,203-206]
Aspecific symptoms	2 (1.2)	[85,166]
Other clinical conditions	46 (28)	[17,22,34,36,54,59,66,84,89,100,102,110,113,124,125,127,133-135,140,155,159,171-174,207-213]
HIV-related opportunistic infections/IRIS	4 (2.4)	[24,26,36,105]

Transplant is surely an event that poses the *Strongyloides* - infected patient at high risk of developing HS/DS. We collected 28/244 (11.5%) cases of HS/DS in transplant patients, of whom 19 (68%) died. A couple of patients who developed hyper infection also had co infection with CMV [21,107]. All the surviving patients received ivermectin, either as single treatment (1 patient) or in combination with albendazole (7) or thiabendazole (1) [29,54,60,71,90,92,94,142,145,150].

HTLV-1 infection is a well known risk factor (sometimes in association with related haematological malignancies), of which we found 24/244 (10%) reports ([214,158,120,159,53,54,143,122,12,111,32,216,77,126,163,114,80,35,164,175,155,116]). Ten of the 24 patients (42%) died. One patient had HTLV-1-HIV co infection [122]; he developed an *E. coli* meningitis but successfully responded to ivermectin, two doses given some days (not specified how many) apart.

**Figure 2:**
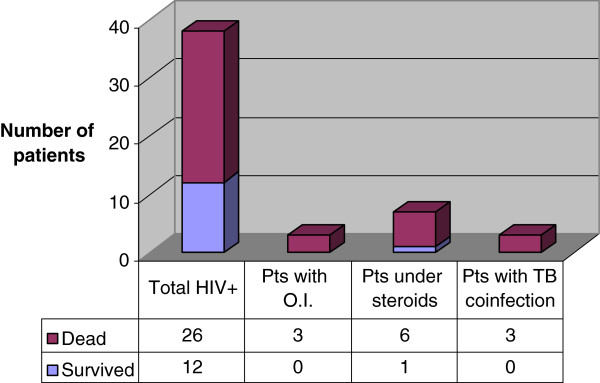
Deaths in the HIV patients subgroup.

We found 38/244 (15%) reports on HIV-positive patients, 26 (68%) of whom died (Figure 2). Seven HIV patients were also receiving steroids for suspected *Pneumocystis jiroveci* pneumonia [24,26], immune reconstitution inflammatory syndrome [105], misdiagnosis of asthma [58], Wegener granulomatosis [36], toxoplasmosis encephalitis [36], cerebral TB with vasculitis [124]; six of them died.

A few reports/case series describe severe strongyloidiasis in patients with alcoholism [178,217] and malnutrition [27,171]. An apparently immunocompetent patient developed hyper infection and died two days after having started therapy with thiabendazole [149]. Unfortunately autopsy was denied.

### Diagnosis

Eosinophilia was present in 55/244 cases (22.5%) overall, and only in 12/73 cases (16.4%) of dissemination. In all cases *S. stercoralis* was found at microscopy examination of biological samples. Serology was performed only in 16/244 patients (6.5%) (Table 2). In a couple of organ transplant recipients, an ELISA test was negative pre-transplant, but resulted positive in the deceased donors (test performed retrospectively) [60,145]. In other two cases serology (ELISA) was negative: a HIV-infected person, who had larvae in stool and sputum [165] and a patient with dermatomyositis, under chronic treatment with prednisone and methotrexate, who died from disseminated strongyloidiasis (larvae found at autopsy in skin, lungs, small and large bowel, gall bladder, vessels of meninges and cervical spinal cord) [100].

**Table 2:** Patients tested with serology: chronic conditions and corticosteroids therapy

**Year**	**Chronic condition**	**Corticosteroids**	**Serology**	**Ref.**
1991	polychondritis	Yes	positive	[89]
1994	COPD	Yes	positive	[57]
1996	HIV	No	**negative**	[165]
2001	none	Yes	positive	[166]
2004	bronchogenic carcinoma	Yes	positive	[160]
2004	none	Yes	positive	[183]
2005	multiple myeloma	Yes	positive	[72]
2005	multiple myeloma	Yes	positive	[204]
2005	nephrotic syndrome	No	positive	[204]
2007	nephrotic syndrome	Yes	positive	[139]
2008	none	Yes	positive	[144]
2008	asthma	Yes	positive	[101]
2009	heart transplant	Yes	**negative**	[145]
2009	lung transplant	Yes	positive	[92]
2010	dermatomyositis	Yes	**negative**	[100]
2011	renal transplant	Yes	**negative**	[60]

Diagnosis was obtained post mortem in 29 cases (12%).

### Therapy

Therapies given were very different in relation to the drugs used and the length of treatment.

In Table 3 we summarize the drugs used. In the “other drugs” group we found mebendazole [9,17,44,48,131,137,181,218], cambendazole [35,36], levamisole [43,199], pyrantel pamoate [75,108], diethylcarbamazine [14].

**Table 3:** Treatments

**Drug**	**Albendazole**	**Ivermectin**	**Thiabendazole**	**Other drugs**
**Used as single treatment**	34	38	55	6
**Deaths among patients treated with single drug**	25/34 (73%)	18/38 (47%)	28/55 (51%)	5/6 (83%)
**Total of patients treated (including combination therapy)**	48	79	60	14

Albendazole was used as a single drug even in recent case reports; since 2008 we found patients treated with albendazole only in reports from Pakistan [203], Romania [217], Taiwan [182], Israel [187], Kuwait [184], Argentina [25], Malaysia [207], Greece [133], Thailand [208].

In most cases the administration route was oral, but due to severe clinical conditions of patients, administration via nasogastric tube, subcutaneous injection (veterinary formulation) and retention enema were used, too.

A patient who developed disseminated strongyloidiasis after an empiric steroid treatment for pruritic rash was treated with albendazole [166]. Only one dose could be given, as the patient died. After his death, a review of his clinical records showed that he had been previously diagnosed with strongyloidiasis and treated with a 3-day course of albendazole; although serology persisted positive and eosinophilia was still present 6 and 12 months after treatment, the patient did not receive any further therapy. Another patient who died from *Strongyloides* hyper infection had never been treated previously, despite a positive serology [101].

### Outcome

The recorded deaths were 153/244 (62.7%). A similar fatality rate was observed in patients with dissemination (50/73 = 68.5%) and with hyperinfection (102/171 = 60%).

All 42 of 244 patients who did not receive any therapy died. Excluding patients treated with combination therapy, we observe that 25/34 (73%) patients treated with albendazole died, while deaths among patients treated with ivermectin and thiabendazole were 18/38 (47%) and 28/55 (51%), respectively.

## Discussion

Considering that a considerable number of case reports are described in non endemic countries, we assume that fatal cases must be quite frequent in endemic countries, although they are not frequently published in the literature.

The main risk factors identified in this review have been reported previously, in particular steroids are frequently the trigger for developing severe strongyloidiasis. Unfortunately it was not possible to extract from the case reports the cumulative dosage and the duration of the corticosteroids treatment. Although the association with steroids should be well known, there are still papers reporting cases of patients under steroids who had not been previously screened for strongyloidiasis. Moreover, we found papers reporting severe strongyloidiasis in patients who were previously diagnosed with the infection but had not received a proper treatment. Once more, the lack of familiarity with strongyloidiasis by health care providers is the weak link in the chain; this is also highlighted by the fact that in 12% of cases the diagnosis was made post mortem. Eosinophil count is often normal in severe strongyloidiasis, hence this test has a limited excluding power.

Serology was not frequently performed. In fact, in case of hyperinfection and dissemination the diagnosis is easily made by direct examination of the biological samples. Serology would be most useful in chronic infections, before hyperinfection and/or dissemination occur, while in patients who are already immune suppressed its sensitivity is probably lower.

Limits in our results are due to incomplete information in the case descriptions. Moreover, cases in which autopsy was not performed sometimes couldn’t allow a proper classification. Actually, in the 65 cases we classified as hyper infections, autopsy was not done, hence it is not possible to rule out dissemination. Moreover, we found the same fatality rate for patients with hyper infection and with dissemination, but a misclassification might have played a role. In fact, we think that from a clinical, practical point of view the distinction between hyper infection and dissemination is not essential, because they’re both severe conditions requiring immediate assessment and care.

In general, the best drug to treat strongyloidiasis is ivermectin which is effective and well tolerated. There are still some concerns about the treatment schedule of the chronic infection, and this is even more debated in case of hyper infection/dissemination. In fact there are no specific guidelines and the case reports we collected outline a Babylon of different therapeutic schemes. Subcutaneous ivermectin (veterinary formulation) has been used on an empiric basis, when intestinal absorption is decreased or the patient cannot swallow tablets. On the other hand, albendazole is still used even as a single drug, although it has been proved to be poorly effective. In some cases, this might be due to the scarce availability of ivermectin in many countries.

## Conclusions

The first step to be done to guarantee an adequate management of infected patients is to avoid a delayed diagnosis. Unfortunately, lack of familiarity with strongyloidiasis by health care providers still seems to be the main cause of delay. A better diffusion of the available information is badly needed, and collaboration among different specialists (oncologists, rheumatologists…) is desirable in order to provide common and adequate protocols for screening and treatment of at – risk patients.

It is mandatory to treat patients in the chronic phase, before HS/DS develop. Patients with possible, previous exposure to the parasite should be screened with serology before corticosteroid treatment, chemotherapy or transplant. Considering the high tolerability of ivermectin, it would be probably worth treating high – risk patients irrespective of the result of the screening test, in order to avoid the potential consequences of a possible false negative result.

Ivermectin is currently the gold standard for treatment of strongyloidiasis, so it is simply no more ethical to use any other drug. Moreover, ivermectin is in the WHO model lists of essential medicines [219], so it should be registered and made available everywhere, particularly in endemic countries.

## Competing interests

The authors declare that they have no competing interests.

## Authors’ contributions

DB searched PubMed, analyzed the data and wrote the manuscript. ARM analyzed the data and critically reviewed the manuscript. AA created the search strategy and analyzed the data. JM, FG and JVDE gave intellectual content and critically reviewed the manuscript. ZB conceptualized the review and critically reviewed the manuscript. All authors read and approved the final manuscript.

## Authors’ information

**Cohemi project study group**: Maurizio Bonati, Francesca Severino, Valeria Confalonieri, Chiara Pandolfini, Zeno Bisoffi, Dora Buonfrate, Andrea Angheben, Marco Albonico, Alessandro Bartoloni, Marianne Strohmeyer, Lorenzo Zammarchi, Jose Muñoz, Robert Pool, Ana Requena-Mendez, Maria Roura, Anita Hardon, Christopher Pell, Peter Chiodini, Juan Moreira, Roberto Sempértegui, Mariella Anselmi, Eduardo Gotuzzo, Maria Alejandra Mena, Hector H. Garcia, Javier Bustos, Saul Santiva, Faustino Torrico,Daniel Lozano, Guido Chumiray Rojas, Teresa Hinojosa Cabrera, Javier Ochoa Morón, Ignacio Abapori Cuellar, Jaime Amorós Suarez, Gianni Tognoni, Alessandra Nicoletti, Elisa Bruno

## Pre-publication history

The pre-publication history for this paper can be accessed here:

http://www.biomedcentral.com/1471-2334/13/78/prepub
